# Left-Sided Hepatic Hydrothorax in Cryptogenic Liver Cirrhosis With Portal Hypertension: A Case Report

**DOI:** 10.7759/cureus.78345

**Published:** 2025-02-01

**Authors:** Duha Shalatouni, Ahmed Alsayed, Abeer Omar, Jamal Sajid

**Affiliations:** 1 Internal Medicine, Hamad General Hospital, Doha, QAT

**Keywords:** ascites, hydrothorax, liver cirrhosis, pleurodesis, transudative effusion

## Abstract

Hepatic hydrothorax is a known complication that occurs in 5-10% of patients with liver cirrhosis and is thought to account for approximately 2% of all pleural effusions. While patients with hepatic hydrothorax typically have ascites, this is not always true.

In this case report, we present a 66-year-old female, known to have liver cirrhosis, who presented with recurrent left-side unilateral pleural effusion without ascites that required frequent therapeutic tapping for symptomatic relief. Her unique presentation made the diagnosis of our case challenging, requiring extensive investigations and diagnostic and therapeutic interventions that all led to a diagnosis of a unique presentation of refractory left-sided hepatic hydrothorax. For better quality of life, pleurodesis via video-assisted thoracic surgery was performed until she was ready for liver transplantation.

Clinicians should remain vigilant about the possibility of hepatic hydrothorax despite the absence of abdominal ascites.

## Introduction

Hepatic hydrothorax (HH) is a pleural effusion that develops in patients with decompensated liver cirrhosis and portal hypertension where cardiac, pulmonary, and pleural diseases are ruled out. The amount of fluid is typically more than 500 mL [1].

HH is a rare condition estimated to occur in approximately 5-10% of patients with liver cirrhosis. Clinical manifestations include dyspnea, cough, pleuritic chest pain, fatigue due to hypoxemia, and abdominal distension [2]. HH commonly occurs on the right side (around 85%), followed by the left side (around 13%), and then bilaterally (around 2%) [3]. Patients with HH are found to have an increased risk of mortality. In one retrospective study that investigated the effect of refractory HH and ascites on survival, the results showed that the one-year mortality of the HH group was 51.06%, and that of the refractory ascites group was 19.15% [4]. Therefore, early recognition and familiarity with available treatment modalities are significant for effectively managing this imperative complication of cirrhosis [5].

In this report, we present a case of refractory left-sided HH in a patient with cryptogenic liver cirrhosis and portal hypertension but without significant ascites.

## Case presentation

A 66-year-old female patient presented to the hospital with a chief complaint of progressive shortness of breath (SOB) over the last week.

Initially, SOB was with exertion; however, at the time of presentation, she had SOB at rest and could not lie flat. She also complained of subjective fever. She did not have chest pain, cough, or upper respiratory tract infection. This was her fourth presentation in the last five months, with the same presentation features, managed by therapeutic drainage, which provided only temporary relief.

She has a past medical history of diabetes mellitus type 2, hypertension, and cryptogenic liver cirrhosis diagnosed in 2018 and was complicated by ascites, grade 4 esophageal varices, and pancytopenia. Her home treatment consisted of 40 mg furosemide once daily, 10 mg propranolol once daily, 100 mg spironolactone once daily, and 250 mg ursodeoxycholic acid twice daily. However, the patient was not compliant with her medications.

Vital signs on admission were as follows: temperature 36.8ºC, heart rate 98, blood pressure 134/74 mmHg, and respiratory rate (RR) 22.

On physical examination, the patient was tachypneic and unable to lie flat, but her oxygen saturation was 96% on room air. On chest examination, she had decreased air entry on the left side with crackles and dullness on percussion. There were no signs of ascites on abdominal examination.

**Figure 1:**
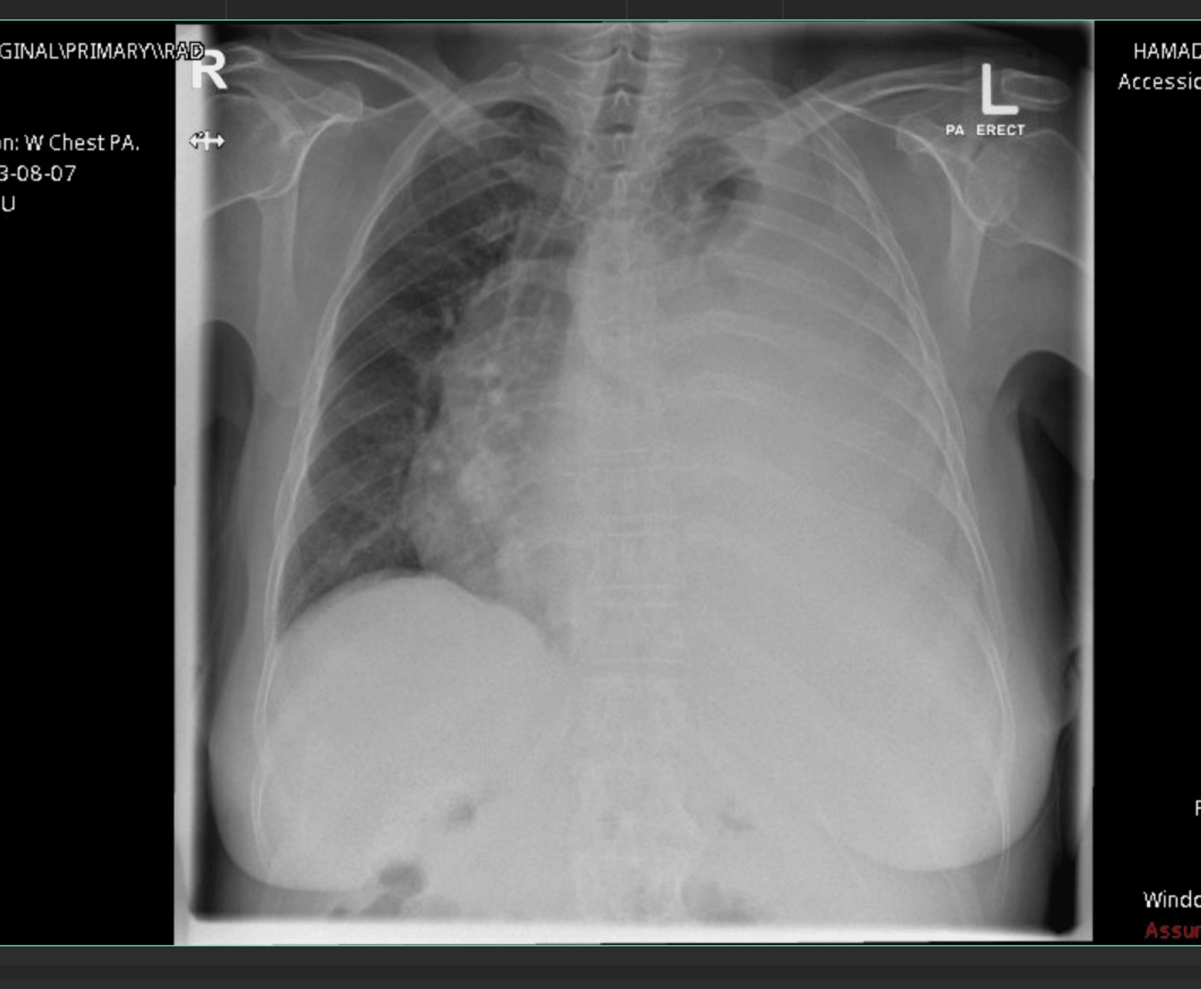
August 7, 2023, on the day of admission

Upon admission, her chest X-ray showed opacification of the left hemithorax, resulting in a mediastinal shift toward the right side with underlying lung atelectasis, while the right lung appeared clear (Figure 1). Her blood tests showed a mild elevation in white blood cells (WBCs) compared with her baseline, thrombocytopenia, elevated international normalized ratio (INR), and low albumin, with normal renal function test (RFT), liver function tests, and C-reactive protein (CRP) (Table 1).

**Table 1:** Lab results upon admission ALT, alanine aminotransferase; AST, aspartate aminotransferase; CRP, C-reactive protein; Hgb, hemoglobin; INR, international normalized ratio; WBC, white blood cell

Detail	Value with units	Normal range
INR	1.6	-
WBC	9.4 × 10^3^/uL	4.0-10.0 × 10^3^/uL
Hgb	7.8 g/dL	12.0-15.0 g/dL
Platelet	42 × 10^3^/uL	150-410 × 10^3^/uL
Albumin level	25 g/L	35-50 g/L
Alkaline phosphatase	125 U/L	35-104 U/L
ALT	16 U/L	0-33 U/L
CRP	11.9 mg/L	0.0-5.0 mg/L
AST	27 U/L	0-32 U/L

Therapeutic thoracocentesis was performed. Cytology and microbiology results were negative, and pleural fluid analysis showed transudative effusion according to Light's criteria (Table 2), which narrows down the differentials to the three most common causes of transudative effusions: heart failure, cirrhosis with ascites, and nephrotic syndrome.

**Table 2:** Pleural fluid analysis BF, body fluid; LDH, lactate dehydrogenase

Detail	Value with units	Normal range
BF type cell count	Pleural	-
Color BF	Yellow	-
Appearance BF	Sl turbid	-
Total nucleated cell BF	275 uL	-
Serum total protein	70 g/L	60-80 m/L
Serum LDH	615 U/L	235-214 U/L
Body fluid LDH	74.0 U/L	-
Body fluid protein	16.5 g/L	-

In most cases of HH, the source of pleural effusion is from abdominal ascites. However, as this patient had no clinical signs of ascites on examination, excluding other causes of unilateral pleural effusion was needed before confirming a diagnosis of HH.

The abdominal ultrasound (US) revealed only mild ascites, with no evidence of ovarian masses indicative of Meigs' syndrome. This was confirmed by abdominal computed tomography (CT). Additional potential causes of pleural effusion were excluded through a cardiac echocardiogram, which showed an ejection fraction (EF) of 54% with no signs of heart failure. No lung or pleural pathology or evidence of superior vena cava obstruction was found on chest and abdomen CT. The thyroid function test was within the normal range.

After ruling out all other differential diagnoses, she was diagnosed with HH without abdominal ascites.

**Figure 2:**
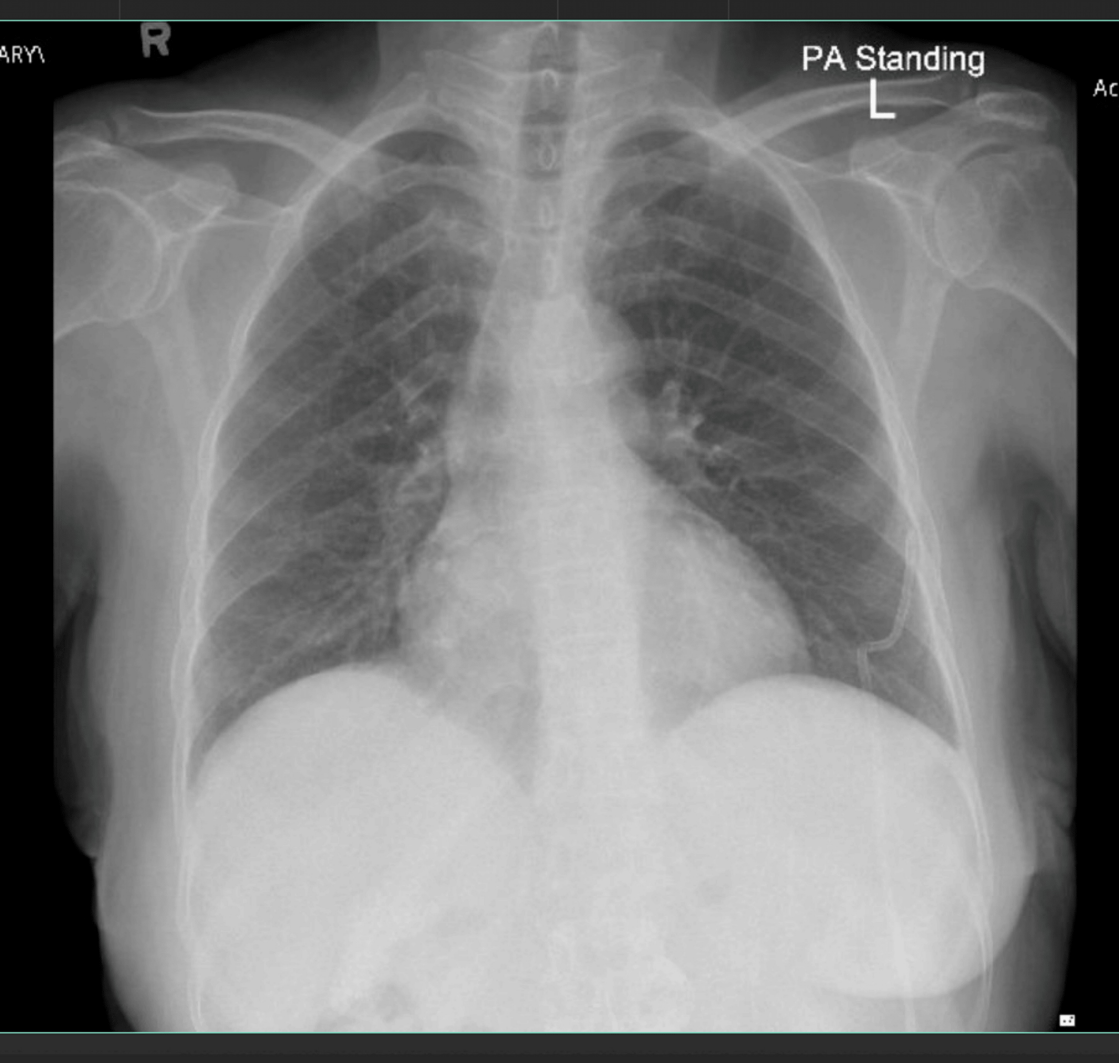
August 16, 2023, six days after chest drain insertion

Initially, the same doses of home medications were resumed: 40 mg furosemide once daily, 10 mg propranolol once daily, 100 mg spironolactone once daily, and 250 mg ursodeoxycholic acid twice daily. A pigtail drain was inserted, and a total of 5,700 mL was drained over a few days. Patient symptoms improved, and chest X-ray revealed that both lung fields and both costophrenic angles were clear and that the left chest drain was in place (Figure 2).

**Figure 3:**
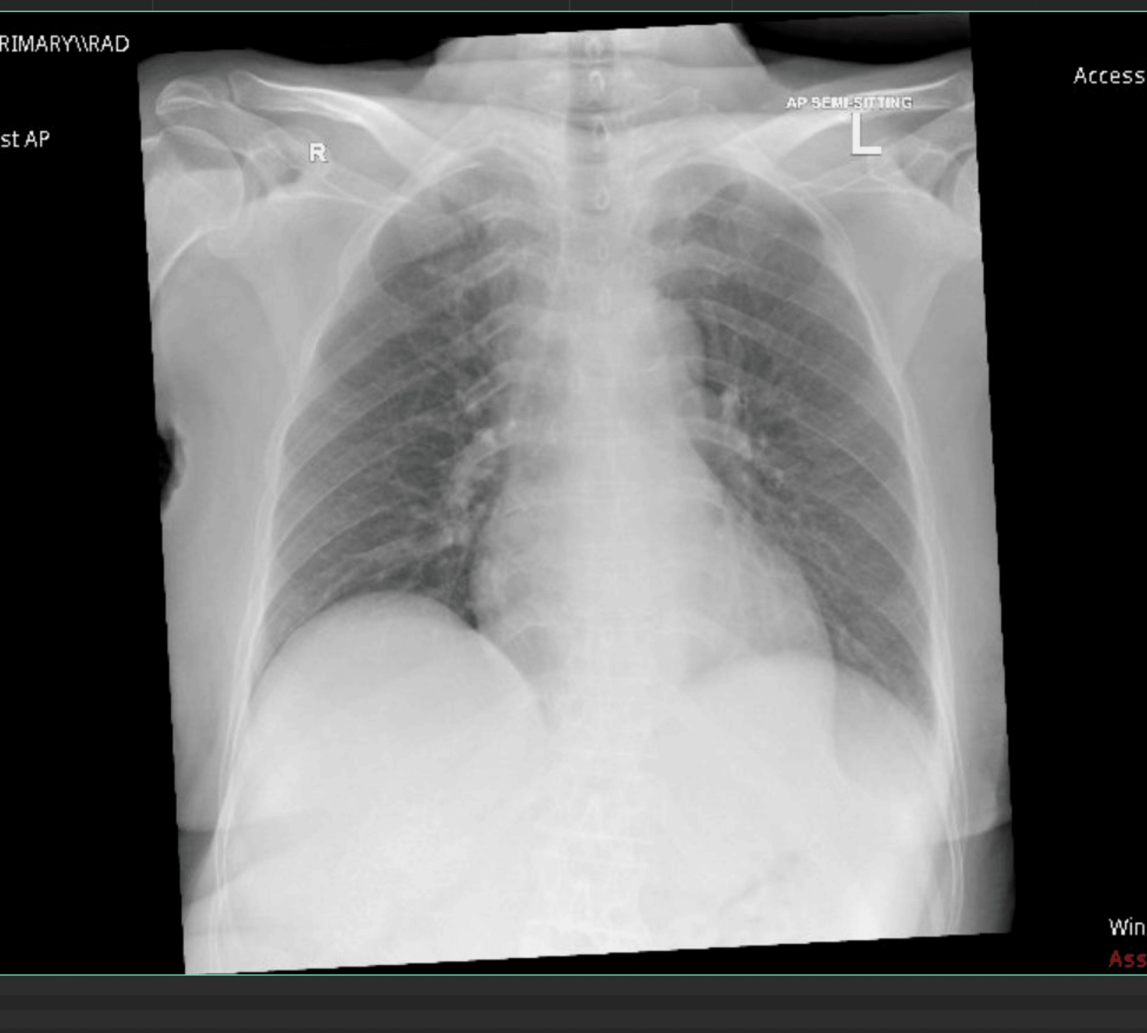
August 26, 2023, six days after the video-assisted thoracoscopic surgery

However, the chest drain still produced approximately 450 mL daily despite increasing the doses of oral spironolactone to 200 mg/day and furosemide to 80 mg/day. To further control rapid fluid accumulation, the chest drain was removed, and chemical pleurodesis was performed via video-assisted thoracic surgery. A repeat chest X-ray six days after surgery (Figure 3) showed no evidence of pleural effusion in the lung.

She was discharged on furosemide (60 mg, oral, daily) and spironolactone (250 mg daily), with regular follow-ups. After following the patient for six months after the pleurodesis, she never presented to the hospital complaining of SOB or requiring chest drain insertion; however, a recent MRI performed during the process of a liver transplant showed massive left pleural effusion with underlying lung collapse and moderate ascites.

## Discussion

Hepatic hydrothorax (HH) is an infrequent consequence seen in individuals with advanced liver disease and develops in just 5-10% of people with end-stage liver disease [6]. Although the exact mechanisms of hepatic hydrothorax are not fully understood, one theory suggests that pleural effusion is derived from the ascitic fluid that enters the chest through defects in the diaphragm because of the negative intrathoracic pressure generated during inspiration as well as increased intrabdominal pressure [7]. These diaphragmatic abnormalities are often less than one centimeter in size, and they are mostly found on the right side (85%), followed by the left side (13%), and both sides (2%) [8]. More hypothetical reasons include hypoalbuminemia, reduced colloidal (oncotic) pressure, and increased azygous vein pressure and flow with ensuing plasma leakage [9].

HH typically coexists with ascites and primarily manifests in the right pleural cavity. There aren't many documented cases of HH that occur without apparent ascites [1,10]. Merely 6% of cases of pleural effusions occupy more than half of the hemithorax. Most cases are mild to moderate. Most reported cases include symptoms such as dyspnea at rest (34%), cough (22%), nausea (11%), and pleuritic chest discomfort (8%). Some patients had no symptoms, and the findings of the radiological examination were incidental [7]. In addition to symptoms, the most often reported clinical signs of hepatic hydrothorax are those related to the consequences of decompensated cirrhosis [1].

It is necessary to rule out other causes of pleural effusion first, including cardiac, pulmonary, pleural, and malignant conditions, before diagnosing HH [7]. For all patients with suspected HH, diagnostic thoracentesis is required to rule out other possible diagnoses and the existence of an infection [5]. Typically, pleural fluid possesses transudate-like properties [11].

An intraperitoneal injection of 99mTc human serum albumin or 99mTc sulfur colloid should be carried out if the diagnosis of HH is uncertain, particularly when the pleural effusion is left-sided and/or ascites is not present. Radioisotope migration into the pleural cavity within a few hours demonstrates contact between the peritoneal and pleural regions [7]. Another diagnostic method to directly detect the underlying diaphragmatic abnormalities is thoracoscopy [5].

Hepatic hydrothorax, especially refractory hydrothorax in particular, poses a challenging therapeutic dilemma, as there are limited treatment options available for these patients. Sodium restriction and diuretic treatment are part of management, and when they fail, liver transplantation remains the most definitive therapy for refractory patients. However, there are other effective strategies for patients who are not candidates for liver transplants or who are awaiting organ availability. These include transjugular intrahepatic portosystemic shunt (TIPS) or video-assisted thoracoscopic (VATS) repair of the diaphragmatic defects (with or without chemical pleurodesis) [5]. Successes rate of chemical pleurodesis is 90%, but it depends on several factors, including the underlying cause, the presence of pleural effusion at the time of the procedure, and the agent used [12].

Recurrent symptomatic malignant pleural effusions have been effectively treated with indwelling tunneled pleural catheters (ITPCs). For patients with HH who do not respond to standard medical care, ITPC can be a suitable course of action [13].

## Conclusions

Hepatic hydrothorax (HH) is associated with high mortality, and available guidelines primarily focus on HH with ascites, leaving a significant gap for cases without ascites or with mild ascites that can not explain the pleural effusion, to rely in management on case reports and expert opinion.

Our case report aims to emphasize the importance of considering hepatic hydrothorax as a differential diagnosis when a patient presents with left-sided pleural effusion in a background of liver cirrhosis, after excluding more common diagnoses. In addition to addressing this gap to improve patient outcomes and reduce risks.
